# An optimized 5/6 nephrectomy mouse model based on unilateral kidney ligation and its application in renal fibrosis research

**DOI:** 10.1080/0886022X.2019.1627220

**Published:** 2019-06-25

**Authors:** Rui-Zhi Tan, Xia Zhong, Jian-Chun Li, Yu-Wei Zhang, Ying Yan, Yuan Liao, Dan Wen, Hui Diao, Li Wang, Hong-Chun Shen

**Affiliations:** a Research Center of Integrated Chinese and Western Medicine, Affiliated Traditional Medicine Hospital, Southwest Medical University, Luzhou, China;; b College of Integrated Chinese and Western Medicine, Southwest Medical University, Luzhou, China;; c Depertment of Nephrology, Affiliated Traditional Medicine Hospital, Southwest Medical University, Luzhou, China

**Keywords:** CKD, 5/6 nephrectomy, renal failure, renal fibrosis

## Abstract

5/6 Nephrectomy (PNx) on rat and mouse mimics renal failure after loss of kidney function in human, and it has been widely used in CKD researches. However, existing methods for PNx model construction present high mortality of animals after modeling due to hemorrhage and infection in or after surgery. Here, we report a novel and highly efficient PNx modeling method to simulate conventional 5/6 nephrectomy, which significantly reduced the mortality of animals and simplified the modeling procedures. In this novel modeling method, we directly ligated the upper and lower poles of left kidney after removal the right kidney 1 week later (l-PNx), which leads to necrosis of ligated upper and lower poles of the kidney and mimics the conventional 5/6 nephrectomy (c-PNx). After modeling 4 and 12 weeks, the serum creatinine, BUN and proteinuria levels were strongly increased in both c-PNx and l-PNx model. Importantly, compared with the c-PNx, l-PNx model present more severe renal fibrosis estimated by Masson staining, IHC and western blotting. The results showed that the protein levels of α-SMA were significantly increased in the kidney of c-PNx and l-PNx models, but more increase was found in l-PNx model. It is noteworthy that, compared with c-PNx model, the survival rate of l-PNx model was markedly increased. In summary, we established a novel and efficient 5/6 nephrectomy model, which can mimic conventional 5/6 nephrectomy to construct a renal fibrosis and renal failure mouse model, that is conducive to mechanism and treatment researches of CKD.

## Introduction

Chronic kidney disease can consecutively progress to end-stage renal disease, and its increasing incidence is a heavy burden on the health systems of countries [1,2]. A large number of studies around the world have demonstrated that the incidence of CKD in the population is increasing with ranges of 10.2% to 20% [3,4]. Therefore, it is extremely important to study the pathogenesis and development mechanism of chronic kidney disease. In this case, the use of CKD animal models to study the mechanism and treatment of CKD is a good research approach. In recent years, several CKD models have emerged, such as glomerulonephritis-induced CKD model (Anti-GBM nephritis [5,6], Anti-Thy 1 nephritis [7,8], and so forth. Partial translation to human disease), aging-induced CKD model [9,10] (Good translation to human disease), radiation-induced CKD model [11,12] (Good translation to human disease), unilateral ureteral occlusion-induced CKD model [13,14] (Partial translation to human disease), and 5/6 nephrectomy-induced CKD model [15–17] (Excellent translation to human disease). Among them, 5/6 nephrectomy (PNx) to rat and mouse is a good simulation of renal failure after loss of kidney function in human [17,18].

The conventional 5/6 nephrectomy model removes one kidney and excises 2/3 of the other kidney 1 week later, which may cause a large amount of kidney bleeding and infection, increasing postoperative animal mortality. A new 5/6 nephrectomy method has recently been reported to simulate a conventional 5/6 nephrectomy by ligation of branches of renal artery [19]. However, this method requires micromanipulation and is not feasible to mouse. Another commonly used 5/6 nephrectomy is to use electrocoagulation to stop bleeding after surgery [20–22]. This method is also to remove the kidney on one side at first, then remove the upper and lower poles of the other kidney, and use electrocoagulation to stop bleeding. Although this method can be used to reduce renal hemorrhage, but it still causes 30–40% of the mice to die, and requires a special electrocoagulation instrument, which increases the cost and complexity of the experiments. Therefore, we hope to optimize the existing 5/6 nephrectomy, reduce renal hemorrhage and infection during surgery, and reduce animal mortality and complexity of the operations. Interestingly, our pre-experiments demonstrated that necrosis of the upper and lower poles of the kidney can be caused by direct ligation on kidney, which offers a possibility to optimize traditional 5/6 nephrectomy. Therefore, this present study was performed to simulate the 5/6 nephrectomy by this simple and easy-operating surgical method, also to verify the effect of this method on renal function and the formation of renal fibrosis, and to explore its possibility of replacement for conventional 5/6 nephrectomy in CKD research.

## Materials and methods

### Unilateral kidney ligation model construction

Eight-week-old male C57BL/6 mice were classified into nine groups: 1-week sham, 1-week 5/6 cut, 1-week 5/6 ligation, 4-week sham, 4-week 5/6 cut, 4-week 5/6 ligation, 12-week sham, 12-week 5/6 cut, and 12-week 5/6 ligation group with 10 mice in each group, and all of these animals were purchased from Chengdu Dossy Experimental Animals Co., Ltd (Chengdu, China), and housed in pathogen-free, temperature-controlled environment with a 12-h/12-h light/dark cycle and allowed free access to food and water. For conventional 5/6 nephrectomy (c-PNx) model, the right kidney was exposed through a flank incision and removed. After a week, the left kidney was exposed and the upper and lower poles were cut away through a flank incision. For control animals (SHAM), each flank of the kidney underwent sham surgery by incising flanks, exposing kidney and suturing each flank. For ligation based 5/6 nephrectomy (l-PNx) model, the right kidney was exposed and removed. After a week, the left kidney was exposed and the upper and lower poles were ligated with 3–0 non-absorbable suture till the diameter of coil was 1/2 of the kidney. Mice in each group were sacrificed at 1, 4, and 12 weeks after surgery, blood, and kidney were collected for laboratory analysis. All animal experiments were carried out according to the guidelines approved by the Animal Ethics Committee of Southwest Medical University. The acceptance number of the application for ethical review of animal research of this study is 20171211009.

### Detection of physiological parameters

The serum electrolytes were detected by automatic biochemical analyzer AU680 (Beckman) at the clinical laboratory of the Affiliated Traditional Medicine Hospital of Southwest Medical University. The blood pressure was detected by intelligent noninvasive blood pressure monitor (BP-2010A, Ruanlong, China). The blood pressure monitor was prepared and preheated in advance, then put the sensor on the tail of the mouse, and the mice were acclimated for 5 min, subsequently, measured the blood pressure in tail, and each mouse was repeatedly measured three times.

### H&E staining

Kidney was fixed in 4% para-formaldehyde and dehydrated in gradual ethanol solution followed by embedding in paraffin wax. Then, the embedded kidneys were cut into 3 μm serial sections. The sections were dewaxed in xylene and rehydrated in ethanol gradients, followed by staining with hematoxylin-eosin (H&E, Beyotime, China) for histological evaluation. Finally, sections were imaged using a Virtual Slide Microscope (VS120-S6-W, Olympus, Japan) at ×40, ×100, and ×200 magnification.

### PAS staining

Kidney was fixed in 4% para-formaldehyde, and after dewaxing and rehydrating like in H&E staining, sections were treated with 1% Periodic Acid for 30 min, subsequently, sections were stained with PAS for 45 min, and the nuclei were stained by Hematoxylin for 1 min. Sections were imaged using a Virtual Slide Microscope (VS120-S6-W, Olympus, Japan).

### Immunohistochemistry

For immunohistochemistry (IHC), the steps of dewaxing and rehydration were similar to HE staining. After goat serum blocking for 1 h, the slices were incubated with anti-mouse antibody against α-SMA (1:100, Ebioscience) and TGF-β (1:100, Cell Signaling Technology) at 4 °C overnight and then combined with a secondary antibody for 1 h (horseradish peroxidase-conjugated goat anti-mouse antibody). At last, the antibody binding was visualized through using DAB kit (ZLI-9033, ZSGB-BIO, China). All sections were imaged using a Virtual Slide Microscope (VS120-S6-W, Olympus, Japan) at ×200 magnification.

### Masson’s trichrome staining

For Masson’s Trichrome Staining, the Masson’s Trichrome Stain kit was used following the product introduction. Briefly, deparaffinize slides before washing with deionized water, followed by staining slides in Weigert’s Iron Hematoxylin Solution for 5 ∼ 10 min, and washing in running warm tap water for 10 min. Subsequently, stain the samples in Ponceau-Acid Fucshin for 5 ∼ 10 min and rinse briefly in deionized water. Then, place slides in 1% Phosphomolybdic Acid Solution for 5 min, Aniline Blue Solution for 5 min and 1% Acetic Acid for 1 min, respectively. Finally, dehydrate samples in 95% and 100% alcohol, clear in xylene and cover with mounting media. All sections were imaged using a Virtual Slide Microscope (VS120-S6-W, Olympus, Japan) at ×200 magnification.

### Western blotting

The protein level of α-SMA, Collagen I and Collagen III in kidney were measured by western blotting. Equal amounts of protein lysates from mouse kidney were resolved in SDS-PAGE gel and transferred to PVDF membrane (Milipore, Billerica, MA). After blocking with 5% BSA for 1 h, monoclonal antibody against mouse α-SMA (1:1000, Ebioscience), Collagen I (1:1000, Santa cruz), and Collagen III (1:1000, Santa cruz) were employed to incubate the membrane at 4 °C overnight. Subsequently, the membranes were incubated with the corresponding secondary antibody (horseradish peroxidase-conjugated secondary anti-mouse antibody, 1:5000; ZSGB-Bio, Beijing, China) at room temperature for 1 h. Antibody binding was visualized using ChemiDoc XRS + system (Bio-Rad, Hercules, CA). Gray intensity of western blot signal band was calculated with Image J 1.47 V software (NIH, Bethesda, MD).

### Serum creatinine and BUN detection

Cr and BUN assay kit (Nanjing Jiancheng Institute of Biotechnology, C011-2, C013-2, Nanjing, China) were used to detect the serum Creatinine and BUN in each group and all operations were followed the instructions.

### Statistical analysis

Data analysis was performed with one-way analysis of variance (ANOVA) test with *post hoc* contrasts by Student–Newman–Keuls test by using SPSS software 21.0 (SPSS, Inc., Chicago, IL). The statistical analysis of survival rate was performed with Log-rank (Mantel-Cox) test by Prism 7.0 software. For all studies, data are presented as mean ± standard deviation. *p* Values less than .05 were considered statistically significant.

## Results

**Figure 1.**
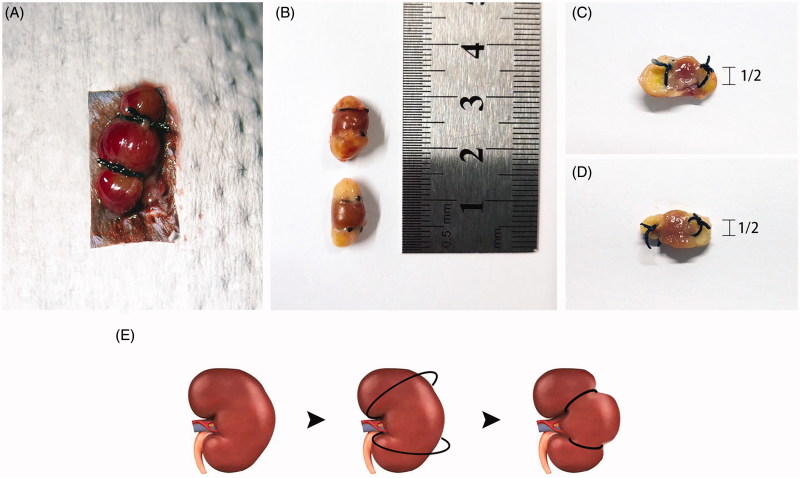
Unilateral kidney ligation leads to necrosis of upper and lower poles of kidney after 1 week. (A) The operation manner of l-PNx model. (B) After 1 week, the upper and lower poles of kidney have been necrotic by ligation. (C,D) Ligation of the upper and lower poles with sutures till the diameter of coil was 1/2 of the kidney. (E) Diagrammatic presentation of l-PNx model.

Unilateral kidney ligation leads to necrosis of upper and lower poles of kidney after 1 week. According to the methods mentioned in the Materials and methods section, we established a novel mouse 5/6 nephrectomy model by excising the right kidney and ligation of the upper and lower poles of the left kidney 1 week later. As shown in Figure 1(A), the upper and lower poles of the left kidney were ligated by 3–0 non-absorbable suture. One week after ligation, the upper and lower poles of the kidney were markedly necrotic, as shown in Figure 1(B). The cross section of the kidney also shows that the kidney is extremely necrotic after 1 week of ligation, and the diameter of ligation coil is half of the kidney when ligating, so that it does not cause bleeding, and can achieve a good ligation effect, as shown in Figure 1(C,D). Figure 1(E) is a diagram of kidney ligation.

**Figure 2.**
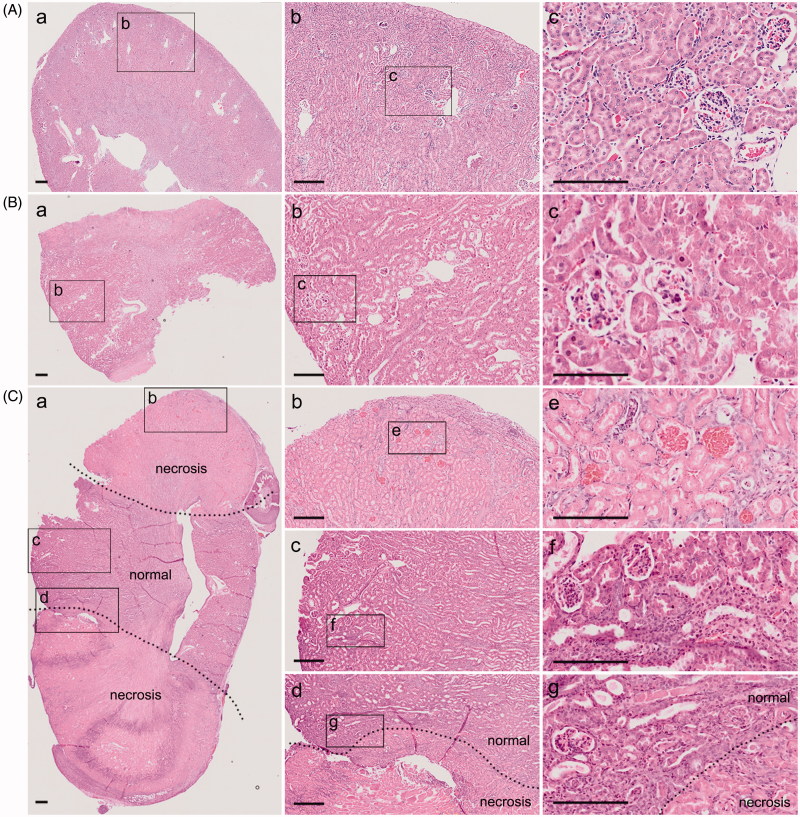
Unilateral kidney ligation simulates conventional 5/6 nephrectomy. (A) H&E staining of kidney in sham group after surgery 1 week ((Aa) is a × 40 image, (Ab) is a magnified image from (Aa) with ×100 magnification, and (Ac) is a magnified image from (Ab) with ×200 magnification). (B) H&E staining of kidney in c-PNx group after surgery 1 week ((Ba) is a × 40 image, (Bb) is a magnified image from (Ba) with ×100 magnification, and (Bc) is a magnified image from (Bb) with ×200 magnification). (C) H&E staining of kidney in l-PNx group after surgery 1 week ((Ca) is a × 40 image, and (Cb) is necrotic portion, Cc is normal portion, and Cd is junction portion with ×100 magnification, (Ce,Cf,Cg) is magnified image from (Cb,Cc,Cd) with ×200 magnification).

Unilateral kidney ligation simulates conventional 5/6 nephrectomy. In order to test whether l-PNx model can mimic the pathological features of c-PNx model, we used the traditional method to establish a 5/6 nephrectomy model, which also removes the right kidney first, and then cut off the upper and lower poles of the left kidney after 1 week. After collecting the kidney samples, we performed the H&E staining to observe the pathological differences in kidneys from sham group (Figure 2(A)), c-PNx group (Figure 2(B)) and l-PNx group (Figure 2(C)). The results showed that there were no pathological differences in the kidneys from c-PNx and l-PNx model. Results from Figure 2 illustrated that with the l-PNx model, upper and lower poles of the kidney undergoing an ischemic necrosis, but the middle portion has no effect, so it can simulate the c-PNx model.

**Figure 3.**
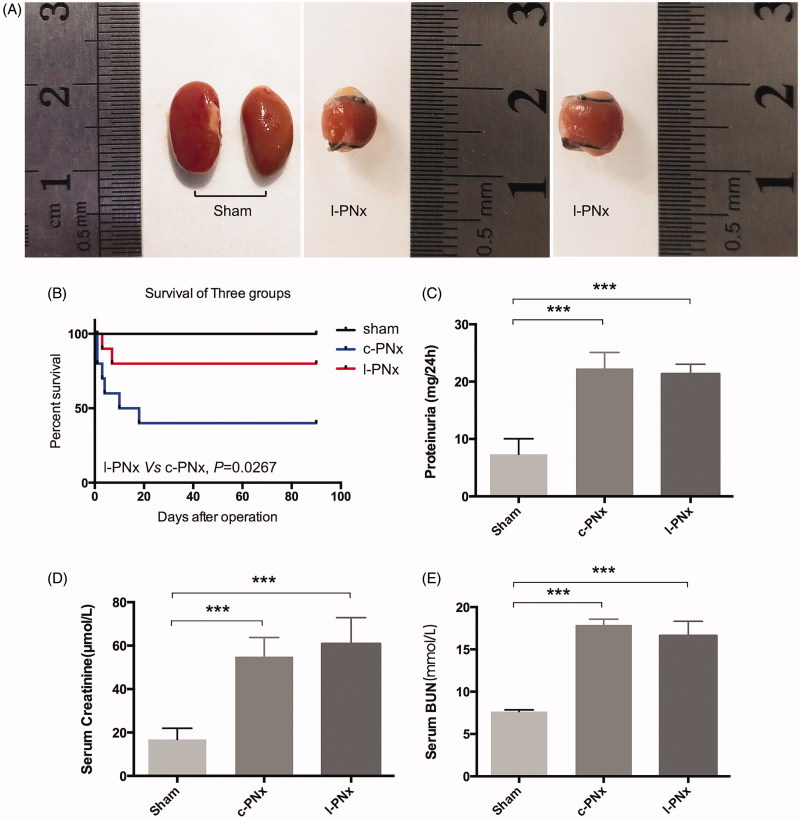
Unilateral kidney ligation increases serum Creatinine, BUN and Proteinuria just the same as c-PNx model, but significantly reduces mortality after 4 weeks. (A) Sham kidney and ligated kidney after surgery 4 weeks. (B) The survival curve of mouse in sham, c-PNx and l-PNx groups. (C) The proteinuria level of each group after surgery 4 weeks. (D) The serum creatinine level of each group after surgery 4 weeks. (E) The serum BUN level of each group after surgery 4 weeks. ***p < .001.

Unilateral kidney ligation increases serum Creatinine, BUN and Proteinuria just the same as c-PNx model, but significantly reduces mortality at 4 weeks after surgery. At the 4th week after modeling, we calculated the survival rate of sham, l-PNx and c-PNx mice, and also collected the kidney tissue, as well as detected renal function, such as serum creatinine and BUN. The results showed that at the 4th week after surgery, the upper and lower necrosis parts of l-PNx model have been almost absorbed, leaving only 1/3 of a single kidney, as shown in Figure 3(A), which has completely simulated the renal morphology of c-PNx model, but the survival rate of postoperative mouse was greatly improved due to reduced bleeding during surgery and simplified surgical procedures. As shown in Figure 3(B), the 4-week and 12-week survival rate of l-PNx model was 80%, and which in c-PNx group was 40%. Renal function test showed that the serum creatinine, BUN and proteinuria of the l-PNx and c-PNx models were significantly higher than those of the sham group at the 4th week after surgery (Figure 3(C–E)), indicating that the l-PNx model was functionally simulated the c-PNx model, but the animal survival rate was significantly improved.

**Figure 4.**
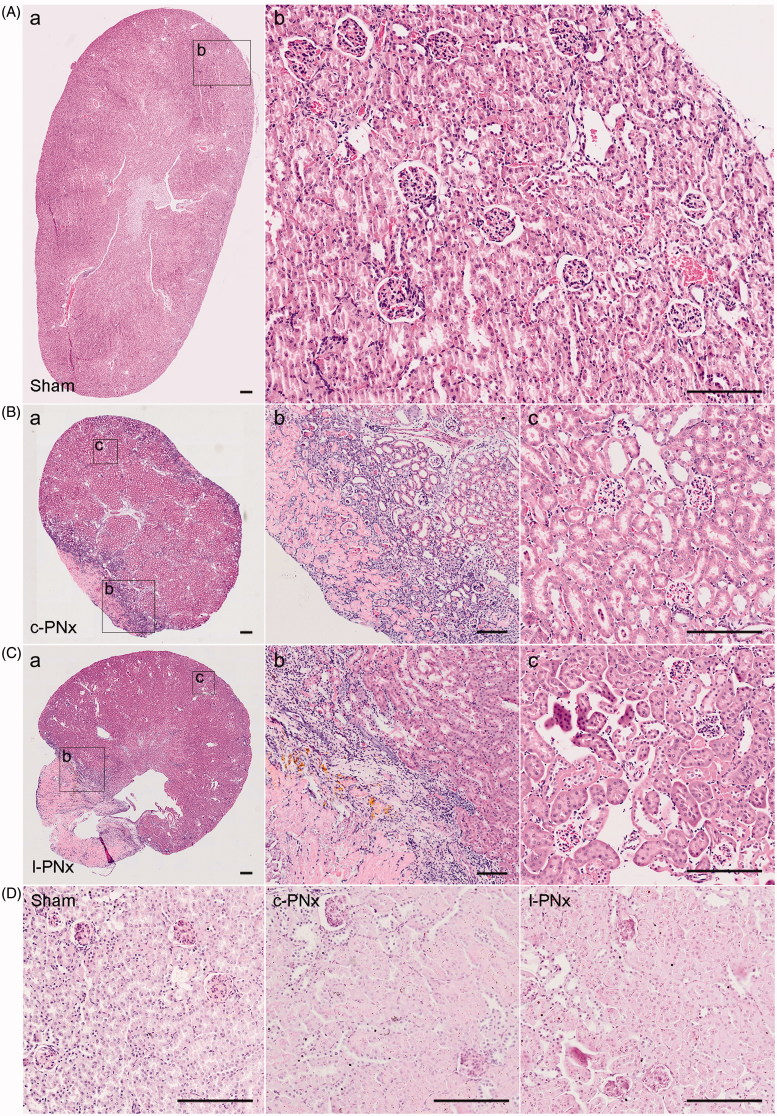
No pathological difference between c-PNx and l-PNx model kidney at 4 weeks post-operation. (A) H&E staining of kidney in sham group after surgery 4 weeks ((Aa) is a × 40 image, (Ab) is a magnified image from (Aa) with ×200 magnification). (B) H&E staining of kidney in c-PNx group after surgery 4 weeks ((Ba) is a × 40 image, (Bb) is a magnified image of junction portion of necrosis and normal from (Ba) with ×100 magnification, and (Bc) is a magnified image of normal portion from (Ba) with ×200 magnification). (C) H&E staining of kidney in l-PNx group after surgery 4 weeks ((Ca) is a × 40 image, (Cb) is a magnified image of junction portion of necrosis and normal from (Ca) with ×100 magnification, and (Cc) is a magnified image of normal portion from (Ca) with ×200 magnification). (D) PAS staining of kidney in each group (×200 magnification).

No pathological difference between c-PNx and l-PNx model kidney at 4 weeks post-operation. We further examined the pathological changes of the kidneys at 4 weeks after modeling. The results showed that the H&E staining results of the kidneys of l-PNx model were not different from those of the c-PNx model. There was no necrosis of renal tubules, no significant shedding of tubular epithelial cells was observed and there was no difference in glomeruli (Figure 4(A–C) present sham, c-PNx and l-PNx kidney, respectively), indicating that the model of l-PNx could mimic the c-PNx model at 4 weeks after surgery. We also performed PAS staining to identify the pathological changes of the kidneys at 4 weeks after modeling, no significant difference was found between each group (Figure 4(D)).

**Figure 5.**
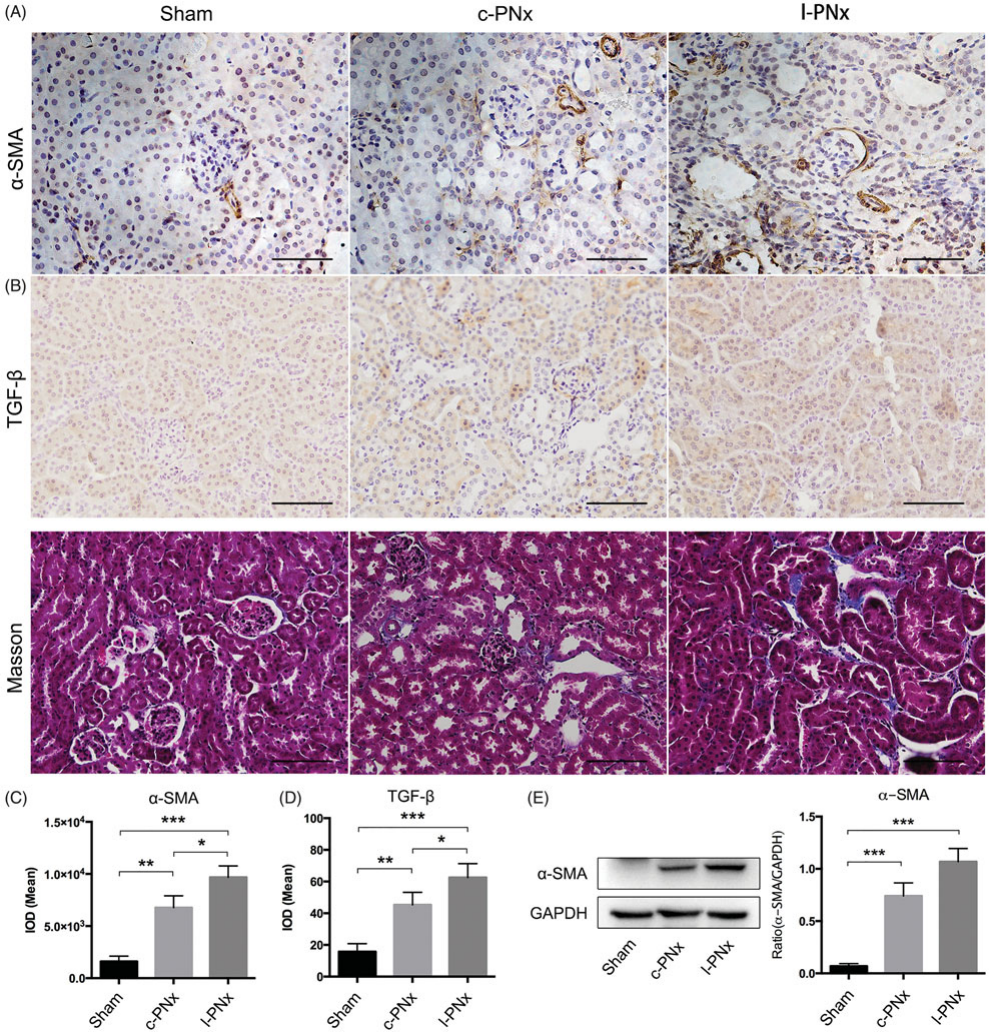
Unilateral kidney ligation based 5/6 nephrectomy can induce kidney fibrosis at 4 weeks post-operation. (A) The IHC results of α-SMA and TGF-β in kidney of sham, c-PNx and l-PNx groups (×200 magnification). (B) Masson staining of kidney in each group (×200 magnification). (C) Mean IOD of α-SMA IHC in each group. (D) Mean IOD of TGF-β IHC in each group. (E) The western blotting result of α-SMA. *p < .05. **p < .01. ***p < .001.

Unilateral kidney ligation can induce kidney fibrosis at 4 weeks post-operation. To detect renal fibrosis in sham, c-PNx and l-PNx model, we performed immunohistochemistry of α-SMA and TGF-β, as shown in Figure 5(A), and the mean IOD of α-SMA and TGF-β IHC were illustrated in Figure 5(C,D). The results showed that l-PNx and c-PNx model can lead to elevation of α-SMA and TGF-β level in the kidneys at 4 weeks after modeling. We further performed Masson staining, as shown in Figure 5(B), the results showed that there were blue fibrosis staining in the kidney of c-PNx and l-PNx models, suggesting that both models have elevated fibrosis. Furthermore, renal fibrosis of the l-PNx model was higher than the c-PNx model. In addition, we examined the protein level of α-SMA by western blotting, as shown in Figure 5(E). The results showed that α-SMA was highly expressed in both models, and its expression in l-PNx model was higher than in c-PNx model. The above results showed that the l-PNx model can simulate the occurrence of renal fibrosis in c-PNx model, and the degree of fibrosis is more serious.

**Figure 6.**
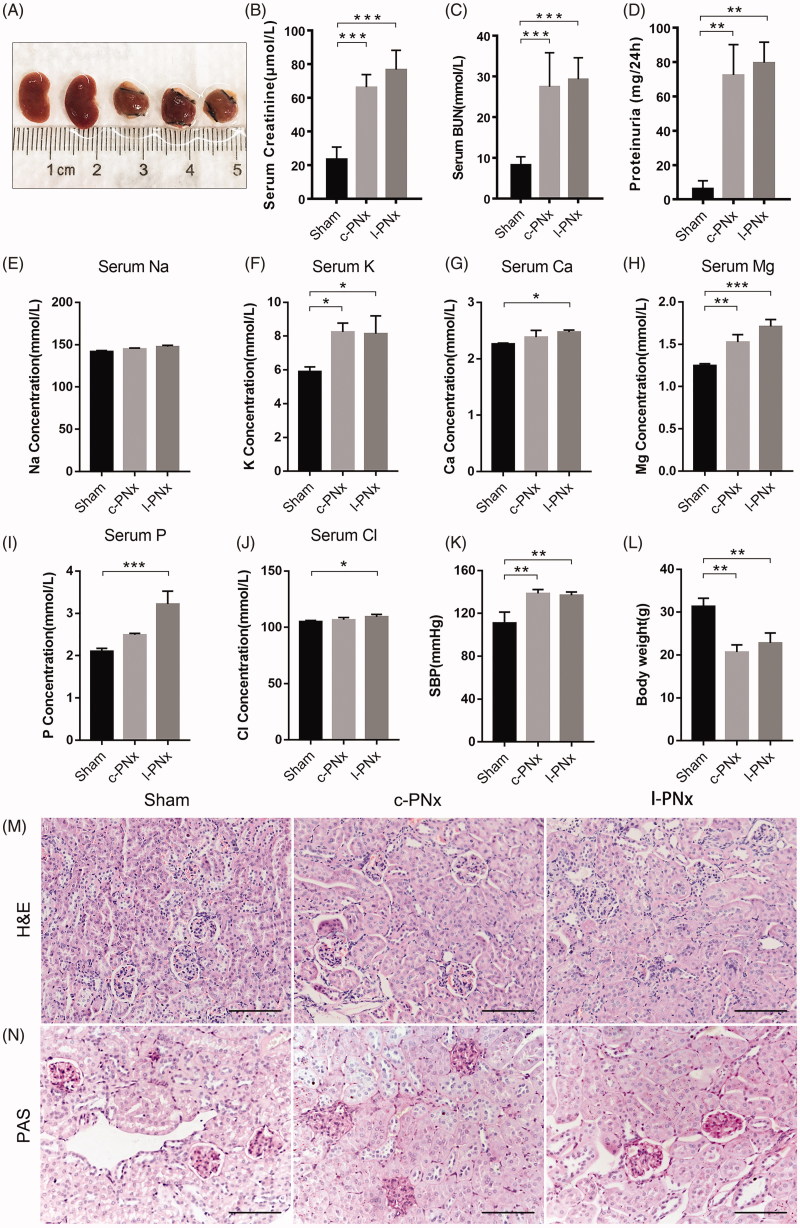
Unilateral kidney ligation mimics the pathological and physiological changes of CKD at 12 weeks post-operation. (A) Sham kidney and ligated kidney after surgery 12 weeks. (B) The serum creatinine level of each group after surgery 12 weeks. (C) The serum BUN level of each group after surgery 12 weeks. (D) The 24 h proteinuria level of each group. (E–J) The serum sodium, potassium, calcium, magnesium, phosphorus and chlorine levels in each group. (K) The blood pressure of each group. (L) The body weight of each group. (M) The H&E staining of kidney in each group (×200 magnification). (N) The PAS staining of kidney in each group (×200 magnification). *p < .05. **p < .01. ***p < .001.

Unilateral kidney ligation mimics the pathological and physiological changes of CKD at 12 weeks post-operation. In order to investigate the pathological and physiological changes in l-PNx model at 12 weeks post-modeling, which is the conventional time point of CKD modeling, we collected mouse kidney and blood samples after 12 weeks of surgery. The results showed that the upper and lower necrosis parts of l-PNx model have been almost absorbed at 12 weeks post-modeling, leaving only 1/3 of a single kidney, as shown in Figure 6(A), which was consistent with the morphology of ligated kidney at 4 weeks post-modeling. The data of renal function tests showed that the serum creatinine, BUN and proteinuria of the l-PNx model were significantly higher than those of the sham groups at the 12th week after surgery (Figure 6(B–D)), indicating that the l-PNx model was functionally simulated the c-PNx model after 12 weeks of modeling. Moreover, we also detected the serum electrolyte, such as, serum sodium, potassium, calcium, magnesium, phosphorus and chlorine levels (Figure 6(E–J)) in each group. The results demonstrated that the serum potassium, calcium, magnesium, phosphorus and chlorine levels in l-PNx model were significantly higher than sham group. Then, we also detected the blood pressure (Figure 6(K)) and body weight (Figure 6(L)) in all groups, the data showed that the blood pressure in l-PNx model was as high as in c-PNx model, which was strongly high than sham group. However, no pathological difference between c-PNx and l-PNx model kidney was observed at 12 weeks post-operation detecting by H&E staining and PAS staining (Figure 6(M,N)). Above data suggested that l-PNx model was an effective CKD mouse model just like the c-PNx model.

**Figure 7.**
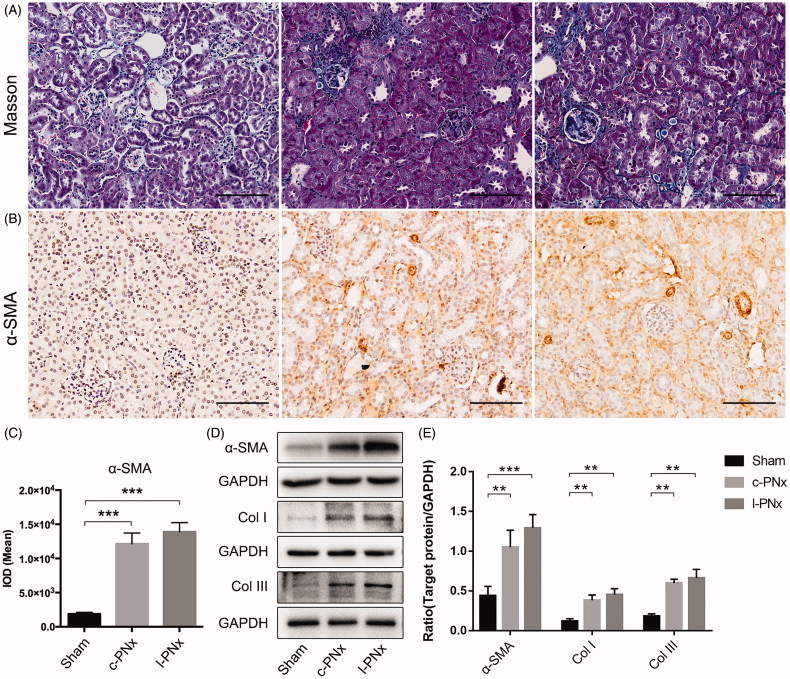
Unilateral kidney ligation based 5/6 nephrectomy induces more seriously renal fibrosis at 12 weeks post-operation. (A) The Masson staining of kidney in each group (×200 magnification). (B) The IHC result of α-SMA in kidney of each group (×200 magnification). (C) Mean IOD of α-SMA IHC in each group. (D, E) The western blotting results of α-SMA, Collagen I and Collagen III. **p < .01. ***p < .001.

Unilateral kidney ligation can induce severely kidney fibrosis at 12 weeks post-operation. In order to investigate the extent of renal fibrosis in l-PNx model at 12 weeks post-modeling, we also collected mouse kidney samples after 12 weeks of surgery and perfomed a series of detection. The results showed that the formation of fibrosis in kidney of 12 weeks post-modeling was markedly increased than in kidney of 4 weeks post-modeling, which was confirmed by Masson staining (Figure 7(A)), IHC of α-SMA (Figure 7(B,C)) and western blot of α-SMA, Collagen I and Collagen III (Figure 7(E)). These findings demonstrated that unilateral kidney ligation based 5/6 nephrectomy can mimic the CKD modeling at 12 weeks post-surgery and induce severely fibrosis in renal interstitium. Notably, the renal interstitial fibrosis in l-PNx kidney was more severe than in c-PNx kidney, suggesting that l-PNx modeling after 12 weeks of surgery was a better method for CKD modeling than c-PNx.

## Discussion

In this present study, we established a mouse model simulating c-PNx model by ligation of the upper and lower poles of the mouse left kidney a week after resection of the right kidney of the mouse. The optimized model showed the same renal function changes and pathological changes as the c-PNx model at 1, 4, and 12 weeks after surgery, but greatly improved the survival rate of postoperative animals. In addition, the model showed a phenotype of renal fibrosis at the 4th and 12th week after modeling, and increased expression of α-SMA in the kidney, which providing a highly efficient 5/6 nephrectomy model for CKD and renal fibrosis research.

Although there are many methods to establish CKD animal models, the 5/6 nephrectomy rat and mouse models are currently the most used renal failure models, which induce renal dysfunction and renal interstitial fibrosis that simulate the characteristics of CKD. Normally, the c-PNx model is established by removal one kidney and excision of the upper and lower poles of the other kidney 1 week later. During the surgery, the kidney is difficult to stop bleeding after the upper and lower poles are removed, and also easy to be infected, that often leads to postoperative death of animals due to hemorrhage or infection. Thus, it requires a large number of modeling to meet the experimental needs and increase the experimental consumption. In response to these problems, a variety of improved 5/6 nephrectomy models have emerged, such as ligation of renal blood vessels leading to partial renal necrosis and simulating 5/6 nephrectomy, but this method requires micromanipulation and is very difficult to operate on mouse, however, sometimes we need to make a 5/6 nephrectomy model on some genetically modified mouse, so the operability of this method is greatly reduced. In view of this, we hope to establish a simple and easy-operating 5/6 nephrectomy method for mouse. After many attempts, we found that the direct ligation of the upper and lower poles of the kidney can lead to necrosis of the upper and lower poles of the kidney, leaving only the middle of the kidney to functionate, which simulates the c-PNx model. This method avoids the risk of hemorrhage and infection caused by the removal of the upper and lower poles of the kidney, significantly improves the survival rate of postoperative animals, and reduces the workload of experimental modeling. After detecting serum creatinine and BUN at 4 and 12 weeks post-modeling, we found the l-PNx model mimicked the renal function changes of c-PNx, in which the creatinine and BUN were largely increased. H&E staining showed no difference in pathological changes between the l-PNx model and the c-PNx model 4 weeks or 12 weeks after surgery. Renal interstitial fibrosis is a typical feature of the CKD model, at the 4th week after surgery, we observed the formation of fibrosis in the renal interstitial in l-PNx and c-PNx model kidney by Masson staining, and more severe fibrosis in the 12th week. Interestingly, the degree of fibrosis in the l-PNx model kidney was higher than that in c-PNx model. This result was also confirmed by immunohistochemistry and western blotting experiments. In the immunohistochemistry experiment, the expression of α-SMA and TGF-β (The fibrosis markers) in l-PNx model was higher than that in c-PNx model, and the western blotting results also showed that the protein levels of α-SMA, Collagen I and Collagen III in l-PNx model kidney was significantly higher than that in the c-PNx model, which demonstrated that the l-PNx model is a better model than c-PNx model for CKD research, especially for renal fibrosis of CKD.

Compared with the previously reported 5/6 nephrectomy model, this l-PNx model can quickly establish a CKD model, and simulate the pathological changes and renal function changes of conventional 5/6 nephrectomy CKD model, which significantly improved the survival rate of animals after modeling. It should be noted that the suture used in the kidney ligation surgery must be a 3–0 or thicker line. If using a thinner line, such as 4–0 line, it will cause kidney rupturing and bleeding, and increase the mortality rate of animals after surgery.

Our study provided a novel optimization method for 5/6 nephrectomy, which was performed by ligating of the upper and lower poles of the kidney by suture line to simulate the renal nephrectomy. The CKD model made by this method may simulate renal fibrosis and failure, and significantly reduced the mortality of postoperative animals, providing an excellent animal model for mechanism and treatment research of CKD disease.
